# Implementation and Evaluation of a Therapeutic Communication Educational Program for Nurses: Protocol for a Mixed Methods Study

**DOI:** 10.2196/65795

**Published:** 2025-06-12

**Authors:** Krista Hoek, Louise Suur, Monique van Velzen, Elise Y Sarton

**Affiliations:** 1 Department of Anaesthesiology Leids Universitair Medisch Centrum Leiden The Netherlands

**Keywords:** therapeutic communication, rapport, periprocedural anxiety, pain, medical education, nursing program, educational program, nursing, implementation, analysis, study protocol, protocol, hospital admission, well-being, patient-centered care, training program, e-learning, therapeutic, mixed method, qualitative, quantitative, emergency treatment

## Abstract

**Background:**

Admission to a hospital can be a very stressful and anxiety-provoking experience, as patients face many unknowns that can compromise their physical and psychological well-being. Patient-centered care necessitates that health care organizations and professionals actively comprehend patients’ experiences and values, for which effective training in communication skills is essential.

**Objective:**

This study will contribute to this field of research by evaluating a blended therapeutic communication training program.

**Methods:**

The training consists of online e-learning that provides insights into important concepts of therapeutic communication, followed by a virtual reality patient-embodied experience shown to help nurses feel what it is like to be a patient themselves during a 1-day didactic training with experiential small groups. Theory on therapeutic communication is taught, focusing on how to use rapid rapport techniques and hypnotic and suggestive language to facilitate empathy. This is combined with practical exercises, ensuring an active learning process. By integrating these diverse blended learning training methods, the program aims to enhance nurses’ communication skills, ultimately improving patient care. Applying the Kirkpatrick model for training evaluation, this prospective study will use a convergent mixed methods study design, integrating both qualitative and quantitative data. Qualitative data will include fieldwork, as well as individual and focus group interviews with the participating nurses. Quantitative data will include questionnaires that include the first two levels of the Kirkpatrick model and that are validated for this purpose. Inclusion started in April 2024, and the therapeutic communication training was scheduled for the first half of 2024.

**Results:**

On February 8, 2024, we received permission from the authorizing body (Institutional Science Committee and NWMO Committee) to start our study. Data collection started in April 2024 and was completed by the end of 2024.

**Conclusions:**

This study will systematically evaluate the effectiveness of therapeutic communication training in the acute admission ward for patients who require emergency treatment. The results will yield insights into the feasibility and acceptance of the implementation of therapeutic communication training among nurses in an acute admission ward in the Netherlands.

**International Registered Report Identifier (IRRID):**

DERR1-10.2196/65795

## Introduction

Admission to a hospital can be a very stressful and anxiety-provoking experience, as patients face many unknowns that can compromise their physical and psychological well-being. Hospitalization exacerbates patients’ emotional reactions and may impede their ability to cope with and adjust to this new environment [1]. Especially in the dynamic and acute setting of the acute admission ward, patients are susceptible to anxiety, stress, and pain [1,2]. In the acute admission ward, many medical procedures, such as invasive exams and surgery, are well known for causing preprocedural anxiety and stress, with a reported prevalence ranging from 11% to 80% among adult patients undergoing surgery [3,4]. Preoperative anxiety is associated with increased postoperative pain, chronic pain, and lower satisfaction scores, potentially by amplifying the body’s stress response and sensitizing the nervous system [5-9].

Landmark studies on patients’ experiences and values describe that anxiety and stress come from the fear of the unknown, the uncertainty of treatments and their side effects, and the loss of control [3,10]. Patient-centered care necessitates that health care organizations and their professionals actively comprehend patients’ experiences and values [11-14]. To establish high-quality and effective communication within this framework, effective training in communication skills is essential. It may assist health care providers in managing anxious and painful patients.

Despite its recognized importance, communication skills are not consistently or systematically taught in medical and nursing education. There is no general agreement or regulation in the educational system on how to teach and learn communication skills. Communication skills seem to be acquired ‘on the job’, greatly depending on implicit contextual factors rather than conventional classes or training [15]. The question arises of how to best teach and learn therapeutic communication skills and how to sensitize its importance. We hypothesize that a real lived experience of a patient could be a powerful learning experience, allowing health care providers to develop a deeper, more empathetic understanding of the patient's perspective [16,17]. Furthermore, we hypothesize that this understanding fundamentally aids in learning therapeutic communication.

Therapeutic communication can be defined as a way of communicating with awareness of and positive influences on contextual factors [18-20]. Spoken words are potent and suggestive tools that can shape future experiences and perceptions. In addition, communication includes paraverbal elements such as voice pitch, pacing, tone, and volume. Nonverbal aspects such as eye contact and body language also play a significant role in shaping perceptions of the receiver. These elements are crucial for establishing rapport between individuals during interactions. Rapport, defined as a close and meaningful relationship with mutual understanding, involves both verbal and nonverbal communication [21,22]. Incorporating elements of therapeutic communication can improve patients’ knowledge and understanding, foster trust, enhance self-care skills, increase treatment adherence, provide comfort, and aid in managing emotions, all of which are essential for health and well-being [23,24].

While various communication training programs exist, there is limited research on the effectiveness of short, blended-learning approaches that integrate virtual reality (VR)-based experiential learning to enhance therapeutic communication. This study aims to address this gap by evaluating the impact of a one-day blended-learning therapeutic communication training program. The training combines e-learning, VR patient-embodied experience, and didactic training with experiential small groups, providing an innovative approach to skill acquisition.

**Figure 1:**
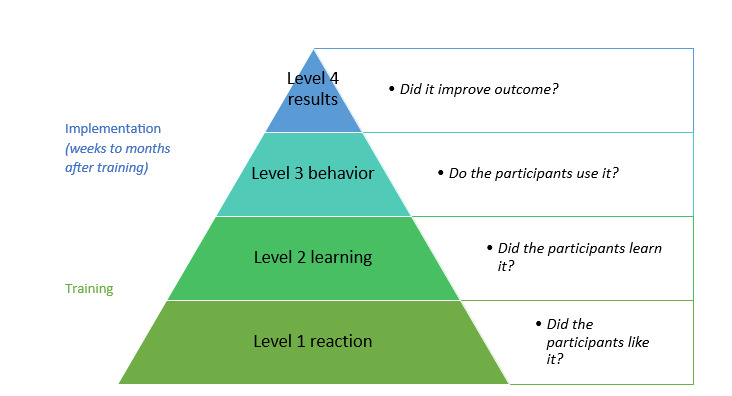
Kirkpatrick four-level training evaluation model.

The main objective of this study is to evaluate the impact of our therapeutic communication training for nurses in the acute admissions ward of a tertiary hospital using the 4-level Kirkpatrick model for training evaluation [25,26], as shown in Figure 1. Our research questions are derived from the first two levels:

Level 1: How do participants *feel* about therapeutic communication training?

Level 2: Did participants *learn* about therapeutic communication?

## Methods

### Setting and Participants

This prospective study will be conducted at the acute admission ward of the Leiden Universitair Medisch Centrum (LUMC) hospital, a tertiary teaching hospital in the Netherlands. In the acute admission ward, patients who need emergent care or treatment can be admitted for a short stay. The protocol was approved by the Institutional Science Committee as well as the non-WMO committee and will adhere to Good Clinical Practice (GCP) regulations.

To be eligible for the study, participants must daily communicate with patients or their relatives as part of their daily duties, speak proficient Dutch (local language) to be able to complete the surveys, and participate in an interview. Nurses leaving the ward within a year were excluded. For the quantitative analysis, nurses of a general ward will also be invited enhancing statistical power with a greater number of participants. In both wards, all eligible nurses were invited to participate in the therapeutic communication training during working hours, and they were invited through email to participate in the study. Participants will not receive any compensation and signed an informed consent form before the start of the study. Pragmatically, the interviews will take place during working hours, inviting nurses on call on the day of the interview. To include broad data, interviews are planned during day and evening shifts on different days of the whole week.

This study will not directly involve patients in the development of the therapeutic communication training, as it was designed based on established literature on patient stress and anxiety as well as previous research on the development and validation of VR-embodied experiences [16,17]. The primary objective of this study is to evaluate the effects of the training on nurses, focusing on their perceptions and learning outcomes. Direct patient involvement in this stage would require a substantial shift in study design and ethical considerations, particularly concerning data collection from patients.

### Study Design

A convergent unblinded mixed method study design in which qualitative and quantitative data are used in multiple phases and merged and analyzed equally for an interactive approach [27]. Qualitative and quantitative investigations will take place before and after the implementation of the therapeutic communication training at the acute admission ward. Additional quantitative data is retrieved at a general ward.

Qualitative research, and in our case ethnography, has an important role in better understanding the social factors influencing medical and scientific routines [28]. A qualitative researcher (CK) will perform fieldwork and immerse themselves in the research population for an extended period, studying insiders’ perspectives, behaviors, and culture. Qualitative outcome measures will include nonparticipatory observations, individual interviews, and focus group interviews with health care providers. During fieldwork, we aim to be naturalistic and nondirective, stepping back from action and minimalizing interference with regular care [29,30].

For quantitative data analysis, data saturation will be pursued [31]. For quantitative outcomes, our pragmatic feasible objective is to include at least 50% of all nurses, which amounts to 20 nurses of the total 40 nurses working in the acute admission ward, and 20 of the 40 nurses in the general ward.

### Intervention

#### Therapeutic Communication Training

The training was developed based on landmark patient-centered studies and included important aspects of lived patient experiences [5-8,19,32-35]. The trainer is a hypnotherapist and anesthesiologist with extensive teaching experience currently working at the LUMC hospital.

The blended therapeutic communication training program includes several components, including online and offline elements, and an overview can be found in Multimedia Appendix 1.

(1) Online e-learning: an online training module covering the basics of therapeutic communication.

(2) VR patient-embodied experience: this immersive experience enables nurses to feel firsthand what it is like to be a patient, enhancing empathy and insight. The VR experience shows the patient journey of a woman who will undergo knee surgery. She was brought into the preoperative holding area, given an IV drip, and driven to the OR. The video ended when the anesthesiologists induced general anesthesia. During the video, the health care providers do not apply therapeutic communication practices, show little empathy or personalized care, and give quite a few negative suggestions during the encounters. A more detailed description of the VR experience can be found in our recent work on patient-embodied VR experiences [16,17].

(3) 1-Day didactic training: this in-person session of a maximum of 10 participants features experiential small-group activities, reinforcing the concepts learned online and in VR. The theoretical foundation of the training focuses on therapeutic communication techniques such as rapid rapport building and strategic word choice using hypnotic language. Techniques such as reframing, mirroring, and positive suggestions are emphasized while avoiding the nocebo effects of negative language and expectations. Practical exercises ensure an active learning process, enabling nurses to apply these techniques effectively in their interactions with patients.

### Quality Assurance

**Figure 2:**
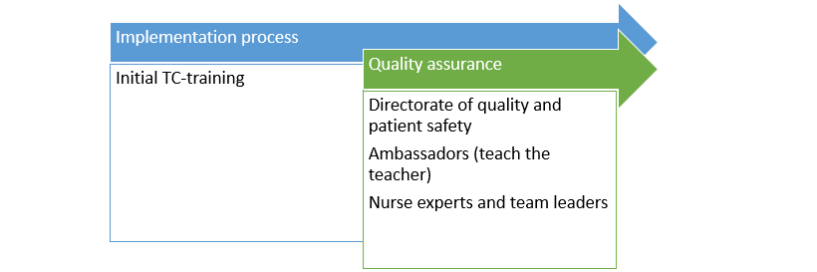
Implementation process.

We hypothesized that the implementation of the therapeutic communication training program would require more than an initial training. To facilitate the full implementation process, nurses, researchers, quality advisors, and communication experts will work together to optimize the implementation process, as shown in Figure 2 and detailed below.

### Directorate of Quality and Patient Safety

To ensure the quality of the learned knowledge of therapeutic communication, the Directorate of Quality and Patient Safety and the Communication Department of the Hospital will be involved throughout this research project. They will meet regularly with the nurse experts and team leaders of the ward to evaluate the implementation process.

### Teaching the Teacher

We hypothesized that it would be necessary to have “therapeutic communication ambassadors” on the ward to be able to answer questions, plan clinical bedside lessons, and perform practical exercises. Recruitment of therapeutic communication ambassadors will start after all nurses in the acute admission ward follow the training. After an initial briefing, an invitation will be sent to participate in “Teach the Teacher” training to become a therapeutic communication ambassador. Team leaders will have the final choice of selection of these ambassadors. This training will be provided by the Radboud Academy, a partner institution of the Nijmegen Academic Medical Centre. During this course, participants will learn to obtain more in-depth knowledge on therapeutic communication, as well as the didactic competencies needed to become an ambassador of the therapeutic communication program.

### Outcomes and Measures

#### Qualitative Outcomes and Measures

We have adhered to Consolidated Criteria for Reporting Qualitative Research (COREQ) criteria (Multimedia Appendix 2) for our qualitative outcomes including, among others, fieldwork, as well as individual and focus group interviews among the participating nurses before and after the training has taken place [36]. The interview guide can be found in Multimedia Appendix 3. Interviews will be audio-recorded, transcribed verbatim, anonymized, and then safely stored.

Focus group interviews are used to interview a small number of demographically similar people in social, psychological, and public health science [37]. During the interviews, nurses will be asked about their perceptions, attitudes, opinions, and views regarding therapeutic communication. Group members are free to talk and interact with each other. Instead of a researcher individually asking group members questions, focus groups will be used to observe group interactions to explore and clarify the beliefs, opinions, and views of participants. During these interviews, the interviewer will take notes and record the discussion for subsequent notetaking to learn from the group. The building of the a priori template and analysis was performed by the principal investigator (KH). For practical reasons, if it is not possible to conduct a focus group interview, individual interviews will be held to gather further data.

The interviews were conducted by research interns (NK and CK), and the researchers (MVV and KH), all with extensive experience in qualitative research. Interviews after the intervention will not be performed by KH, as she is the trainer of the therapeutic communication trainer, and this might induce biases. All interviewers introduced themselves initially and did not know the participants before the commencement of the study.

#### Quantitative Outcomes and Measures

**Figure 3:**
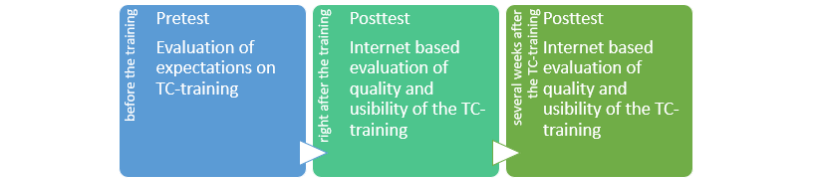
Data collection of quantitative data.

Quantitative outcomes include questionnaires on current knowledge of therapeutic communication, as well as validated training analysis questionnaires following the Kirkpatrick analytical model [26]. Data collection will be conducted as shown in Figure 3.

#### Kirkpatrick Model Level 1 – Reaction: What are Participants’ Reactions to Therapeutic Communication Training?

This questionnaire will be sent out after the training, including themes such as educational goals, trainees’ preparation, the use of new teaching and learning methods, the use of learning tools, teaching environment, teacher assessment, and teacher characteristics on a 5-point Likert scale from very weak to excellent and can be found in Multimedia Appendix 4. [25].

#### Kirkpatrick Model Level 2 – Learning: Knowledge of Therapeutic Communication

For the purpose of this study, a standardized Nursing Education Program Implementation and Analysis (NEPIA) questionnaire was developed to evaluate knowledge of therapeutic communication. This questionnaire included five multiple-choice questions, with one or more correct answers, and can be found in Multimedia Appendix 5. This questionnaire was conducted and validated by a pilot of 10 nurses to evaluate the clarity of the questions. Their responses will not be taken into account in further analysis. This questionnaire aims to respond to the second level of the Kirkpatrick model: “Learning.” What is the impact of therapeutic communication training on participants’ self-efficacy regarding therapeutic communication?

### Data Analysis

All data will be kept in password-protected files with limited user access on protected hospital network drives and collected in the electronic data capture system Castor EDC. For quantitative data analysis, SPSS 25 (IBM Corp) for Windows will be used. Chi-square tests, independent *t* tests, and Mann‒Whitney U tests will be used to test differences before and after training.

Qualitative analysis will be performed using template analysis [38] in ATLAS.ti (Lumivero). Initial themes for the interviews can be found in Multimedia Appendix 3. During the initial coding, a critical reflection will be performed (KH, MVV). If needed, themes are modified, or additional themes are added to ensure that the themes fit the data and that the data are not selectively analyzed to fit the themes. The initial analysis provided information for further participant selection and coding. Performing iterative data collection and analysis to guide decisions will improve the dependability of the results [39]. The template will be further developed by applying it to the full data set. The building of the a priori template and analysis of the interviews will be performed by the principal investigator (KH). We decontextualize the data to identify descriptions and cluster them into discrete categories. Overall, we aim to structure these experiences, and additional changes might be made to the template; more interviews might be needed. Qualitative data will be retrieved while pursuing data saturation; that is, until we are empirically confident, additional data will not lead to any new emergent input [31,40].

### Ethical Considerations

The protocol was approved by the Institutional Science Committee and received a waiver from the institutional review board. The study will adhere to GCP regulations. Written informed consent was obtained from all participants. Participants received no financial compensation. Data will be anonymized.

## Results

On February 8, 2024, we received permission from the authorizing authorities (Institutional Science Committee NWMO Commissie divisie 1) to start our study. Data collection started in April 2024 and concluded by the end of 2024. We expect to publish our results in 2025.

## Discussion

### Overview

This study evaluates the effectiveness of a one-day blended-learning therapeutic communication training for nurses, integrating e-learning, VR patient-embodied experience, and didactic small-group training. Using the Kirkpatrick evaluation framework, we aim to assess whether this structured format enhances nurses’ communication skills, specifically measuring their reaction (Level 1) and learning outcomes (Level 2). We anticipate that the training will be feasible, well-received, and effective in improving nurses’ understanding and application of therapeutic communication techniques. If successful, this study could demonstrate the value of short, immersive training models in clinical education, particularly in fast-paced hospital environments.

Existing research underscores the importance of therapeutic communication in reducing patient anxiety and pain perception; however, most training programs are time and resource-intensive and often lack immersive experiential components. Our one-day format, enriched with VR-based patient embodiment, addresses these gaps by providing a scalable, high-impact intervention. Previous studies on therapeutic communication training have not consistently incorporated technology-driven experiential learning, making this approach unique in its accessibility and potential for broader hospital-wide adoption.

A key strength of this study is its mixed-methods design, ensuring a comprehensive evaluation of both subjective participant experiences and measurable learning gains. In addition, the blended-learning approach, including VR, offers a realistic, patient-centered perspective that may enhance the long-term retention of communication skills.

Another strength of this study is the opportunity to attend the training during working hours, enabling all nurses to participate. The team leaders of both wards will make an effort to accommodate this in the scheduling.

A limitation of this study is that only short-term learning outcomes are assessed, leaving a long-term impact on patient care remaining to be evaluated.

### Dissemination

We intend to present our results within our hospital and advocate for its implementation in other wards, and if possible, throughout the hospital. To enhance the dissemination process, we will use social media platforms and in-house press releases. Furthermore, we aim to publish the results of this study in appropriate peer-reviewed, open-access journals to ensure they are accessible to clinicians, researchers, educators, and other relevant stakeholders within health care organizations, including patients. Furthermore, dissemination through social media and institutional press will support a wider engagement within health care communities.

### Future Implications

In the case of proven effectiveness of therapeutic communication training and implementation, future research should be conducted using level 4 of the Kirkpatrick model for training evaluation: the improvement of patient outcomes. Finally, our results may be useful for other health care institutions and implementation researchers when planning or implementing therapeutic communication skills training.

### Conclusion

This study systematically evaluates a short, immersive therapeutic communication training program by using blended-learning methods, including VR-based patient-embodied experiences, we aim to determine its feasibility and effectiveness in improving communication skills. If successful, this training model has the potential to enhance patient care, influence hospital-wide training initiatives, and serve as a blueprint for scalable communication education programs in health care settings.

## Appendix

Multimedia Appendix 1 Therapeutic communication training.

Multimedia Appendix 2 COREQ checklist.

Multimedia Appendix 3 Interview guide.

Multimedia Appendix 4 NEPIA (Nursing Education on Patient Interaction Assessment) questionnaire – Kirkpatrick model, level 2.

Multimedia Appendix 5 Course evaluation using Kirkpatrick model, level 1.

## Abbreviations

COREQ: Consolidated Criteria for Reporting Qualitative Research
GCP: Good Clinical Practice
LUMC: Leiden Universitair Medisch Centrum
NEPIA: Nursing Education on Patient Interaction Assessment
VR: virtual reality

## Data Availability

The datasets used and/or analyzed during this study are available from the corresponding author upon reasonable request. The original audio files were unavailable due to privacy restrictions.
