# Supplementary material for: Implementation and Evaluation of a Therapeutic Communication Educational Program for Nurses: Protocol for a Mixed Methods Study
Source: JMIR Res Protoc. 2025 Jun 12;14:e65795. doi: 10.2196/65795 (PMC12203028; doi:10.2196/65795)
Supplement: Multimedia Appendix 1 [file resprot_v14i1e65795_app1.docx]

**Therapeutic communication training**

The training will take place between April and August 2025, one to three times per month. Two additional training days will be scheduled for any participant who was not able to follow the training on the planned date.

The TC training will consist of the following:

->E-learning: this e-learning will be available with a personalized link 2 weeks prior to the physical training. It takes about 30-45 minutes to complete the e-learning. The e-learning deploys key insights of therapeutic communication. It encompasses the explanation of important terminology such as placebo, nocebo, negative and positive suggestions, reframing, and *rapport*.

->VR-embodied experiences. There will be 2 VR-embodied experiences available and shown on the day of physical training. The description and validation of these VR experiences can also be found in our earlier published work [16,17].

->physical training with a first part of theoretical knowledge and frameworks, landmark research. This is followed by hands-on training using mirroring rapport techniques and applying therapeutic communication skills with reframing in small groups.

Timeline:

E-learning: 30-45 minutes

Physical training: 5.5 hours (with 45 minutes pause included), and two VR experiences (each around 12 minutes).

### Abbreviations

COREQ: Consolidated Criteria for Reporting Qualitative Research

e-learning: electronic learning

NEPIA: Nursing Education on Patient Interaction Assessment

TC: therapeutic communication

VR: virtual reality
