# Supplementary material for: Implementation and Evaluation of a Therapeutic Communication Educational Program for Nurses: Protocol for a Mixed Methods Study
Source: JMIR Res Protoc. 2025 Jun 12;14:e65795. doi: 10.2196/65795 (PMC12203028; doi:10.2196/65795)
Supplement: Multimedia Appendix 3 [file resprot_v14i1e65795_app3.docx]

# Interview guide

**Topic List for Before the Training**

| **Topic** | **Question** |
| --- | --- |
| **Demographics (Round of questions - all at once)** | - What is your name? - How old are you? - How long have you been a nurse/medical professional? |
| **Pain** | - What do you typically use to evaluate a patient's pain? How do you 'see' that a patient is in pain? - Is this a standard at LUMC? - Follow-up: What do you think about this? - What do you think are the most effective methods to assess pain? - What is the role of doctors in assessing/treating pain? Do they do things differently? - What are your experiences with the Central Pain Team? |
| **Registration** | It happens that the registration of NRS scores is not filled in, especially for the second measurement after a high NRS score. Why is this? Does it ever happen that a pain score does not match how a patient seems to experience the pain? How do you handle this? |
| **Anxiety** | - What do you typically use to assess a patient's anxiety? How do you 'see' that a patient is anxious? - Is there a standard at LUMC (similar to pain scores, but for anxiety)? What do you think about this? - How do you deal with an anxious patient? - What do you think are the most effective methods to assess (to 'see') anxiety? - What is your role in assessing/treating anxiety? What is the role of the doctor? Is there a difference? |
| **TC (Not yet completed)** | - What do you already know about the TC course? - Do you know why the TC course is being introduced? - What are your expectations of this course? - What would you like to learn during the course? |

**Topic List after the Training**

| **Topic** | **Question** |
| --- | --- |
| **Demographics (Round of questions - all at once)** | - What is your name? - How old are you? - How long have you been a nurse/medical professional? - Have you completed the TC training? |
| **TC Training** | - What did you think of the TC training? - To what extent do you think you can apply TC communication? Do you have an example? - Did the training meet your expectations? (Explain why/why not) - Do you need additional training at this time? |
| **Pain** | - During the TC training, it was discussed to evaluate pain and anxiety with a comfort score (NRS comfort), with a checkbox: does the patient need more pain relief. Do you agree with this? - Do you have any other suggestions to improve pain registration? |
| **Free** | - Do you have any other points regarding pain, anxiety, and therapeutic communication that you would like to discuss? |
