# Supplementary material for: Implementation and Evaluation of a Therapeutic Communication Educational Program for Nurses: Protocol for a Mixed Methods Study
Source: JMIR Res Protoc. 2025 Jun 12;14:e65795. doi: 10.2196/65795 (PMC12203028; doi:10.2196/65795)
Supplement: Multimedia Appendix 4 [file resprot_v14i1e65795_app4.docx]

# NEPIA questionnaire – Kirkpatrick model, level 2:

| **Question 1: Which statement is correct?**  a. Pain leads to an increase in inflammatory markers in the blood. b. The brain can open or close pain gates and thus modulate pain. c. A high degree of stress leads to the suppression of the entorhinal cortex, thereby reducing pain perception. d. Shifting attention can increase pain perception.  **Correct: A, B** The brain can open or close pain gates and thus modulate pain. An increase in inflammatory markers is a consequence of stress, not pain. These are very closely related. However, not every (minor) pain prick will cause increased inflammatory markers. How the brain recognizes and interprets pain depends on various factors and complex mechanisms. Pleasant stimuli (e.g., a gentle touch) can close pain gates (gate control), and the brain can also open pain gates, as seen in the feeling of a 'broken heart.' A high degree of fear and stress leads to the activation of certain areas in the brain (entorhinal cortex, insula, and anterior cingulate gyrus), enhancing pain perception. Studies using functional MRI have shown that visual and somatosensory stimuli have pain-modulating functions. Simply put: shifting attention results in less pain.  **Question 2: You are administering a painkiller. Which action will ensure the medication works best? Choose the best answer.**  a) Administer the medication without providing any additional information. b) Say that this is a very effective medication and the patient will likely feel better. c) Say that you see the patient is in pain and this will help. d) Say that you don’t think this medication will work well against the pain.  **Correct: B** Say that this is a very effective medication and the patient will likely feel better. Here, you are giving two positive suggestions: you trust the medication, and you indicate that the patient will feel better. The third answer is less effective because it focuses on the patient's pain, emphasizing a negative feeling, which is unnecessary.  **Question 3: What promotes coping-stimulating behavior? Choose the best answer.**  *Coping: the way someone deals with problems and stress* a. Distraction techniques b. Lots of empathy c. Commentary d. Apology  **Correct: A** Distraction techniques. Distraction techniques promote coping-stimulating behavior. Conversely, too much empathy, commentary, or an apology reduces coping-stimulating behavior because these focus on negative feelings.  **Question 4: Which statement is correct? Choose the best answer.**  a. Empathy always has a positive effect on a patient’s comfort. b. Implicit factors, such as the color or temperature of the environment, affect a patient's experience. c. Anxiety is a detrimental mechanism that activates limbic structures. d. I should always give a warning before inserting an IV.  **Correct: B** Implicit factors, such as the color or temperature of the environment, affect a patient's experience. Atmosphere can be influenced unnoticed by background noises, smells, and colors. The third answer is incorrect because anxiety is not always detrimental. Although anxiety activates limbic structures and triggers the sympathetic autonomic nervous system, causing physiological effects like tachycardia and tachypnea, these are stress responses. The last answer is incorrect because a warning can cause anticipatory stress and increased pain. For example, when inserting an IV, it's better to ask the patient to keep their arm still or consider distracting them by asking a question at the moment of insertion (e.g., what did you have for dinner last night?).  **Question 5: Which statement about the placebo effect is correct?**  a. The placebo effect only works mentally. b. Placebo effects can be harmful. c. In a randomized study, there are no placebo effects. d. Placebo effects can be unconscious.  **Correct: D** Placebo effects can be unconscious. There are implicit or explicit placebo effects, with implicit effects being unconscious. Placebo effects can have biochemical and physiological impacts. Even in randomized studies, there can be (implicit) placebo effects. |
| --- |
