# Supplementary material for: Implementation and Evaluation of a Therapeutic Communication Educational Program for Nurses: Protocol for a Mixed Methods Study
Source: JMIR Res Protoc. 2025 Jun 12;14:e65795. doi: 10.2196/65795 (PMC12203028; doi:10.2196/65795)
Supplement: Multimedia Appendix 5 [file resprot_v14i1e65795_app5.docx]

# Course evaluation using Kirkpatrick model, level 1:

| Improving the quality of continuing education requires your constructive and insightful comments and recommendations. Please help us provide effective training conditions by answering the following questions accurately.  Name of the training course: Basic Therapeutic Communication Training (TC-training)  Date of the course: [Enter the date here]  Please fill in your answer by choosing between:  1: very poor/little  2: somewhat poor/little  3: sufficient  4: somewhat good/much  5: very good/much   \| **Instructor Assessment** \| **excellent** \| **Very good** \| **Intermediate** \| **weak** \| **Extremely weak** \| \| --- \| --- \| --- \| --- \| --- \| --- \| \| Academic proficiency of the instructor \|  \|  \|  \|  \|  \| \| Lecturing method and the ability to transfer the concepts to learners \|  \|  \|  \|  \|  \| \| Ability of the instructor in class management \|  \|  \|  \|  \|  \| \| Use of active teaching methods and engaging the learners \|  \|  \|  \|  \|  \| \| Ability to respond to the ambiguities and questions of learners \|  \|  \|  \|  \|  \| \| Frequency of using practical examples during teaching \|  \|  \|  \|  \|  \| \| **Course content assessment** \| **excellent** \| **Very good** \| **Intermediate** \| **weak** \| **Extremely weak** \| \| Effectiveness of the contents of the course in increasing your knowledge \|  \|  \|  \|  \|  \| \| Relationship between training course and your organizational needs \|  \|  \|  \|  \|  \| \| Up-to-datedness of the contents of the course \|  \|  \|  \|  \|  \| \| Quality of teaching in the training course \|  \|  \|  \|  \|  \| \| **Course support assessment** \| **excellent** \| **Very good** \| **Intermediate** \| **weak** \| **Extremely weak** \| \| Your satisfaction with the duration of the course \|  \|  \|  \|  \|  \| \| Desirability of educational location and environment \|  \|  \|  \|  \|  \| \| Brightness and light in the classes \|  \|  \|  \|  \|  \| \| Ventilation and adequacy of cooling/heating system \|  \|  \|  \|  \|  \| \| Catering and reception \|  \|  \|  \|  \|  \| \| Treatment of authorities toward you \|  \|  \|  \|  \|  \| \| **Overall satisfaction** \| **excellent** \| **Very good** \| **Intermediate** \| **weak** \| **Extremely weak** \| \| Satisfaction of the quality of workshops \|  \|  \|  \|  \|  \| \| Satisfaction of the way of conducting workshops \|  \|  \|  \|  \|  \| |
| --- | --- | --- | --- | --- | --- | --- | --- | --- | --- | --- | --- | --- | --- | --- | --- | --- | --- | --- | --- | --- | --- | --- | --- | --- | --- | --- | --- | --- | --- | --- | --- | --- | --- | --- | --- | --- | --- | --- | --- | --- | --- | --- | --- | --- | --- | --- | --- | --- | --- | --- | --- | --- | --- | --- | --- | --- | --- | --- | --- | --- | --- | --- | --- | --- | --- | --- | --- | --- | --- | --- | --- | --- | --- | --- | --- | --- | --- | --- | --- | --- | --- | --- | --- | --- | --- | --- | --- | --- | --- | --- | --- | --- | --- | --- | --- | --- | --- | --- | --- | --- | --- | --- | --- | --- | --- | --- | --- | --- | --- | --- | --- | --- | --- | --- | --- | --- | --- | --- | --- | --- | --- | --- | --- | --- | --- | --- | --- | --- | --- | --- | --- | --- |
